# Some statistical memes which sound correct but aren't quite: Application to the analysis of observational databases used in learning health systems

**DOI:** 10.1002/lrh2.10219

**Published:** 2020-02-12

**Authors:** Gregory P. Samsa

**Affiliations:** ^1^ Department of Biostatistics and Bioinformatics Duke University Durham North Carolina

**Keywords:** learning health systems, observational studies, randomized trials, statistics

## Abstract

We consider four memes, correct within the context of randomized trials but requiring modification for the analysis of the observational databases typically associated with learning health systems: (a) the right answer always requires randomization; (b) a bigger database is always a better database; (c) statistical adjustment always works if based on a large enough database; and (d) always make a formal adjustment when testing multiple hypotheses. The rationale for these memes within the context of randomized trials is discussed, and the memes are restated in a fashion that is consistent with learning health systems.

## 1 INTRODUCTION

Learning health systems (LHSs) primarily use observational data to (among others) support personalized medicine and systems improvement.^1^ The databases in question can range from very large, to very small, to everything in between. For example, large databases can support personalized medicine by helping to identify which subpopulations might particularly benefit from which interventions. At a much smaller scale, learning can be based on a quality improvement paradigm traceable to Deming.^2^ At a large scale, LHSs intersect with “big data,” whereas at a smaller scale, LHSs intersect with quality improvement. A LHS can potentially have some elements of randomization (or quasi‐randomization) embedded within it: For example, the firm system (whereby separate teams care for similar groups of patients, thus facilitating assignment of interventions across teams and their subsequent comparison) was an early application of that idea.^3^ Nevertheless, a LHS is primarily about the creative use of observational data.

In contrast to the use of observational data in a LHS, the dominant (but not exclusive) paradigm in the general medical literature is the randomized trial (RT). Learning from observational data is both similar to, and different from, learning from RTs. Certainly, the basic principles of statistics hold in both cases. Nevertheless, their application depends on context. This commentary discusses four memes, entirely correct when applied to RTs, but requiring modification when applied to the observational databases used in LHSs. As a practicing biostatistician responsible for collaborating with, and teaching statistics to, change agents within an LHS, I encounter these memes (sometimes explicitly stated, sometimes not) on a regular basis. The goal of this commentary is not to generate novel statistical results but rather to help clarify thinking about some concepts which can become confused.

## 2 METHODS

This paper is organized around four memes commonly encountered in practice. For each meme, we discuss why it is sound when applied to an RT and then propose a more nuanced version appropriate for the LHS context.

## 3 RESULTS

### 3.1 Meme 1: The right answer always requires randomization

The biomedical literature contains an extensive discussion of the advantages and disadvantages of RTs, which are designed to evaluate drugs, devices, procedures, models of care, etc. The evaluation could be based upon efficacy (ie, impact under ideal conditions), effectiveness (ie, impact under usual conditions), or something in between. Various groups (eg, Guyatt et al^4^) have declared RTs to be the “highest level of evidence,” with observational studies (OSs) occupying a lower rung.

The rationale for placing RTs at the highest level of evidence is both theoretical and empirical. The primary methodological rationale for preferring RTs is that properly executed randomization reduces bias by (approximately) balancing the groups being compared on both measured and unmeasured variables associated with the outcome. The results of empirical comparisons between RTs and OSs are mixed. Some studies^5^, ^6^ found that RTs and OSs yielded similar conclusions, while others^7^ found that RTs tend to have more conservative estimates of treatment effects. Examples of RTs yielding different conclusions than OSs include antiarrhythmic agents^8^, ^9^ and hormone replacement therapy.^10^, ^11^

RTs are tools which can only be applied to questions involving efficacy. Many important questions do not pertain to efficacy—for example, the level of nausea that patients typically experience during chemotherapy—and are best answered using observational databases. So, at the very least, the meme might be tentatively modified to “the best study design for estimating efficacy is a RT.”

Even if the goal is to assess efficacy, RTs are not always possible. Sometimes investigators lack equipoise, implying that it is unethical to randomize patients into the group thought to be receiving an inferior treatment. Moreover, even when possible, RTs are not always realistically feasible. They can be expensive and time‐consuming and thus might not be worth the expenditure of resources, especially within areas of medicine where treatments are being rapidly developed and improved. Perhaps, then, the rule of thumb could be modified to “when realistically feasible, a RT is always the best design for assessing efficacy.” (Especially, when this rule is accompanied by the corollaries “RTs are not always realistically feasible” and “the results of an efficacy trial executed under ideal conditions might not apply to my particular circumstances.”)

RTs typically estimate the average impact of the intervention and are usually underpowered to detect effects within subgroups. Large observational databases can provide important information about the differential impact of an intervention on specific patients. In the absence of randomization, this information is not necessarily definitive but can be useful nevertheless.

This suggests a final, and more nuanced statement of meme 1: “Randomization is a tool which works well in some circumstances and poorly in others. If realistically feasible, RTs are the best way to assess the overall efficacy of an intervention. Observational databases can provide important insights about which patients might benefit the most from an intervention, and also about the likely impact of an intervention within my specific context. Non‐efficacy questions typically require other study designs.”

In practice, critics of LHS sometimes implicitly adopt the posture “RTs are scientific, OSs are not.” In fact, both are “scientific,” albeit in different ways.

### 3.2 Meme 2: A bigger database is always a better database

The meme that “bigger is better” seems so intuitively obvious as to not even require a moment's thought. In fact, the underlying statistical principle—equally applicable to RTs and OSs—is that the larger the sample size the more precise the results. However, when evaluating an intervention, the considerations include not just precision but also bias. Increasing sample size increases precision but has no impact on bias.^12^ RTs are designed to minimize/eliminate bias, and so more patients imply a “better” (ie, more precise) estimate of treatment effect, centered on the correct value. On the other hand, OSs can have biased estimates of treatment effects (see meme 3)—if so, increasing the sample size will yield an answer that is more precise but no less wrong.

To more accurately state meme 2: “large databases yield more precise estimates than smaller ones, but increasing the size of a database has no effect on bias.”

In practice, meme 2 tends to be propagated by uncritical advocates of “big data” and often goes hand in hand with meme 3.

### 3.3 Meme 3: Statistical adjustment always works if based on a large enough database

In the RT setting, two estimates of intervention efficacy are usually presented: an “unadjusted” version whose only predictor variable is study group and an “adjusted” version which includes additional “covariates.” One reason for covariate adjustment is to increase precision: By taking an advantage of covariates which help to predict the outcome, the overall level of noise in the outcome is reduced, and thus, the signal is estimated more precisely. Another potential reason for covariate adjustment is to account for subtle imbalances between study groups, particularly among small subpopulations of interest. Whether covariate adjustment is required/appropriate in this context is a subject of debate.^13^, ^14^ Considering the former reason for adjustment, the argument is essentially the same as for meme 2: Covariate adjustment has no impact on bias (which for a RT is assumed to be minimal/none), increases precision, and thus is beneficial with no ill effects.

Contrasting RTs and OSs, for a RT, randomization assures that on average, both the measured covariates and the unmeasured covariates are (approximately) balanced. In contrast, for an OS, neither the measured covariates nor the unmeasured covariates are necessarily balanced, and either imbalance could induce bias. Measured covariates are candidates for statistical adjustment. For example, the comparison of surgical complication rates across facilities typically adjusts for observed variables such as patient age and gender. The result is assertions such as “even though the patient populations differed between the facilities being compared we adjusted for differences in case mix using a predictive model derived from a large database, and thus the adjusted differences in outcome are attributable to real differences between the facilities.” (Implicitly: “it is sufficient for a predictive model to be derived from a large database.”)

The above assertion relies on the size of the database used to develop the adjustment model in order to be persuasive. However, if that database is missing important predictors (ie, “unmeasured covariates”), the results of the model could still be biased. For example, if no measure of disease severity is available, or if the measure of disease severity which can be derived from the observed variables is inadequate, the results will nevertheless be biased against those facilities treating more complicated cases. More generally, failure to include a covariate in a predictive model induces bias if the two conditions for “confounding” both hold: (a) the covariate is associated with the outcome; and (b) the covariate is associated with the predictor of interest.^15^ Neither of these conditions depends upon the size of the database.

To more accurately state meme 3: “When working with observational databases case‐mix adjustment works best when the predictive model includes the most important factors affecting the outcome variable, and when those factors are well‐measured. If those important factors are missing or poorly measured bias can result, regardless of sample size.”

In practice, meme 3 tends to be propagated by uncritical advocates of “big data.”

### 3.4 Meme 4: Always make a formal adjustment for testing multiple hypotheses

More than one analysis of an observational database has tested multiple hypotheses and, indeed, more than one manuscript describing such analyses has received a review to the effect of “you must always make a formal adjustment for multiple testing, such as dividing p = 0.05 by the number of tests which were performed.”

This meme is based upon a basic principle of statistics. In plain English: “whether or not the intervention being studied is efficacious, the more statistical tests that are performed the more likely it is that some of these tests will have p < 0.05.” A corollary is “be suspicious of statistically significant results when many tests were performed.”

The question, however, is what the data analyst should do in response to this general principle.^16^ The point upon which most statisticians agree is that context matters. Among others, it matters whether there are many statistically significant results or just a few. It matters whether the results are consistent with the underlying science (recognizing that investigators excel in creating scientific explanations after the fact). It matters whether the results are consistent with one another. Perhaps, it matters whether the statistical tests in question were specified ahead of time.

For RTs, especially those which are used in regulatory submission, an additional consideration is social control of the investigators and the need to enforce standardization. With money and reputation at stake it simply is not prudent to allow the investigators to engage in data dredging, selectively report the most statistically significant results, declare a drug to be efficacious, and then to later discover that their conclusion was overly optimistic. Accordingly, a comprehensive written statistical analysis plan must be developed ahead of time and then assiduously followed. This analysis plan will typically identify a small number of tests as critical. The interpretation of these tests is designed to avoid false positive findings—for example, by applying methods such as the “Bonferroni correction” of dividing 0.05 by the number of tests.

In LHS applications, however, much of the value of the databases in question is that they can be reused in multiple ways, and to answer multiple questions. Applying a formal correction for the number of statistical tests (even if the number of tests could be accurately counted) runs counter to the logic of LHSs. For a LHS, considering the possibility of false positive results remains important. What differs is that this issue is not typically addressed through formal adjustment of *P* value thresholds but instead through the recognition that the observational databases used in LHSs allow discovery to take place with the anticipation of subsequent validation.^17^

To more accurately state meme 4: “always consider the possibility that statistically significant findings are falsely positive—whether the response includes a formal adjustment for multiple testing should depend upon context.”

Meme 4 is typically propagated by those whose statistical training has failed to distinguish between a general principle and its implementation.

## 4 DISCUSSION

Table 1 summarizes the memes in question—both an original version accurate for RTs and a restatement which also applies to the OSs which are the lifeblood of LHSs. In every case, “always” has been eliminated from the original statement of the meme.

**Table 1:** Memes and their restatement

Meme	Restatement
Meme 1: The right answer always requires randomization	Randomization is a tool which works well in some circumstances and poorly in others. If realistically feasible, RTs are the best way to assess the overall efficacy of an intervention. Observational databases can provide important insights about which patients might benefit the most from an intervention, and also about the likely impact of an intervention within my specific context. Non‐efficacy questions typically require other study designs.
Meme 2: A bigger database is always a better database.	Large databases yield more precise estimates than smaller ones, but increasing the size of a database has no effect on bias.
Meme 3: Statistical adjustment always works if based on a large enough database	When working with observational databases case‐mix adjustment works best when the predictive model includes the most important factors affecting the outcome variable, and when those factors are well‐measured. If those important factors are missing or poorly measured bias can result, regardless of sample size.
Meme 4: Always make a formal adjustment for testing multiple hypotheses	Always consider the possibility that statistically significant findings are falsely positive. Whether the response includes a formal adjustment for multiple testing should depend upon context.

The intention of this commentary was not to build a rhetorical straw man and then set fire to it nor to criticize specific individuals, instead it was to reflect on some misconceptions we have encountered while teaching and practicing statistics within the context of LHSs.

Although not necessarily amenable to scientific testing, it might be hypothesized that the cause of the disconnect between the literature, which is nuanced, and the memes, whose initial statements are overly prescriptive, is twofold. First, not all change agents within a LHS have received deep training in statistics. Second and particularly relevant to those whose training is limited to introductory statistics courses, such courses typically use RTs as their use case. This, in combination with the classification of RTs as the highest level of evidence (for estimating efficacy), can induce an overly “RT‐centric” viewpoint, as illustrated by memes 1 and 4 (ie, “the right answer always requires randomization” and “always make a formal adjustment for testing multiple hypotheses”). The original statement of memes 2 and 3 (ie, “a bigger database is always a better database” and “statistical adjustment always works if based on a large enough database”), while not necessarily RT‐centric, does hold for RTs but not OSs. These memes are most typically propagated by uncritical advocates of big data.

In addition to attempting to clarify some misconceptions, and without attempting a comprehensive literature review, this commentary also points readers to various papers which can provide an entrée into a more detailed discussion of the content of the memes.

## CONFLICT OF INTEREST

The author declares that he has no conflicts of interest.
